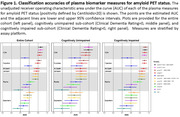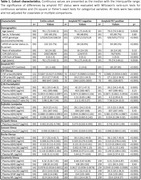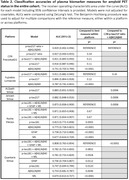# Head‐to‐head evaluation of leading blood tests for amyloid pathology

**DOI:** 10.1002/alz.095506

**Published:** 2025-01-09

**Authors:** Kellen K. Petersen, Suzanne E. Schindler, Duygu Tosun, Benjamin A. Saef, Leslie M. Shaw, Yan Li, Ziad S. Saad, Gallen Triana‐Baltzer, Michael Baratta, Erin G. Rosenbaugh, Anthony W. Bannon, William Z. Potter, the Alzheimer’s Disease Neuroimaging Initiative (ADNI), and the Foundation for the National Institutes of Health (FNIH) Biomarkers Consortium Plasma Aβ and Phosphorylated Tau as Predictors of Amyloid and Tau Positivity in Alzheimer’s Disease Project Team

**Affiliations:** ^1^ Washington University in St. Louis, St. Louis, MO USA; ^2^ Washington University in St. Louis School of Medicine, St. Louis, MO USA; ^3^ Department of Radiology and Biomedical Imaging, University of California, San Francisco, San Francisco, CA USA; ^4^ Washington University in St. Louis, Saint Louis, MO USA; ^5^ Dept of Pathology & Laboratory Medicine, University of Pennsylvania, Perelman School of Medicine, Philadelphia, PA USA; ^6^ Washington University in St. Louis, School of Medicine, St. Louis, MO USA; ^7^ Johnson & Johnson Innovative Medicine, San Diego, CA USA; ^8^ Johnson and Johnson Innovative Medicine, San Diego, CA USA; ^9^ Takeda Pharmaceuticals International, Cambridge, MA USA; ^10^ Foundation for the National Institutes of Health, North Bethesda, MD USA; ^11^ AbbVie Inc., North Chicago, IL USA; ^12^ Highly qualified expert, Philadelphia, PA USA

## Abstract

**Background:**

Blood tests that accurately determine the presence of amyloid pathology are critically needed. Compared to amyloid PET and CSF tests, blood tests are less expensive, less invasive, more accessible, and highly scalable. The Foundation for the National Institutes of Health (FNIH) Biomarkers Consortium evaluated the accuracies of leading AD blood tests in classifying amyloid PET status.

**Method:**

Assays from C_2_N Diagnostics (Aβ42, Aβ40, p‐tau217, p‐tau217 ratio [phosphorylated to non‐phosphorylated tau at position 217]), Fujirebio Diagnostics (Aβ42, Aβ40, p‐tau217), ALZPath (p‐tau217), Janssen (p‐tau217), Roche Diagnostics (Aβ42, Aβ40, p‐tau181, GFAP, NfL), and Quanterix (Aβ42, Aβ40, p‐tau181, GFAP, NfL) were run on plasma samples from ADNI participants with corresponding florbetapir‐PET scans. Unadjusted logistic regression models were constructed for each measure or combination of measures from each assay platform. The classification accuracies of the plasma biomarker measures for amyloid PET status (Centiloids>20) were assessed with receiver operating characteristic area under the curve (AUC) analyses and compared using DeLong’s tests.

**Result:**

The cohort included data from 392 individuals with a median age of 78.1 years; 193 (49.2%) were female; 132 (33.7%) were *APOE ε*4 carriers; 191 (48.7%) were amyloid PET positive; and 192 (49.0%) were cognitively impaired (Clinical Dementia Rating>0), (**Table 1**). Models with high classification accuracies for amyloid PET status included the C_2_N p‐tau217 ratio + Aβ42/Aβ40 (AUC 0.929 [95% confidence intervals 0.902‐0.956]) and Fujirebio p‐tau217 + Aβ42/Aβ40 (0.911 [0.882‐0.940]), (**Table 2**). The AlzPath p‐tau217 (0.885 [0.851‐0.920]), Janssen p‐tau217 (0.882 [0.848‐0.916]), Roche p‐tau181 + Aβ42/Aβ40 + GFAP + NfL (0.873 [0.838‐0.909]), and Quanterix p‐tau181 + Aβ42/Aβ40 + GFAP + NfL (0.808 [0.763‐0.854]) models had lower accuracies than the C_2_N p‐tau217 ratio + Aβ42/Aβ40 model (p = 0.0006, 0.0006, 0.0006, and <0.0001, respectively), (**Figure 1**). In 122 individuals with CSF Roche Elecsys p‐tau181/Aβ42 data, the classification accuracy for amyloid PET status was 0.915 (0.864‐0.967).

**Conclusion:**

In this head‐to‐head study, logistic regression models of C_2_N p‐tau217 ratio + Aβ42/Aβ40 and Fujirebio p‐tau217 + Aβ42/Aβ40 were among the most accurate in classifying amyloid PET status. The classification accuracies of the blood tests for tau PET status, brain atrophy status, and clinical dementia symptoms will also be assessed.